# Long-term functional and radiographic outcomes in 243 operated ankle fractures

**DOI:** 10.1186/s13047-015-0098-1

**Published:** 2015-08-25

**Authors:** S. M. Verhage, I. B. Schipper, J. M. Hoogendoorn

**Affiliations:** MC Haaglanden, Department of Surgery, Postbus 432, 2501 CK The Hague, The Netherlands; Department of Trauma-surgery, Leiden University Medical Center, Leiden, The Netherlands

**Keywords:** Ankle, Bone fracture, Trimalleolar fracture, Patient outcome assessment

## Abstract

**Background:**

Large comparative studies that have evaluated long-term functional outcome of operatively treated ankle fractures are lacking. This study was performed to analyse the influence of several combinations of malleolar fractures on long-term functional outcome and development of osteoarthritis.

**Methods:**

Retrospective cohort-study on operated (1995–2007) malleolar fractures. Results were assessed with use of the AAOS- and AOFAS-questionnaires, VAS-pain score, dorsiflexion restriction (range of motion) and osteoarthritis. Categorisation was determined using the number of malleoli involved.

**Results:**

243 participants with a mean follow-up of 9.6 years were included. Significant differences for all outcomes were found between unimalleolar (isolated fibular) and bimalleolar (a combination of fibular and medial) fractures (AOFAS 97 vs 91, *p* = 0.035; AAOS 97 vs 90, *p* = 0.026; dorsiflexion restriction 2.8° vs 6.7°, *p* = 0.003). Outcomes after fibular fractures with an additional posterior fragment were similar to isolated fibular fractures. However, significant differences were found between unimalleolar and trimalleolar (a combination of lateral, medial and posterior) fractures (AOFAS 97 vs 88, *p* < 0.001; AAOS 97 vs 90, *p* = 0.003; VAS-pain 1.1 vs 2.3 *p* < 0.001; dorsiflexion restriction 2.9° vs 6.9°, *p* < 0.001). There was no significant difference in isolated fibular fractures with or without additional deltoid ligament injury. In addition, no functional differences were found between bimalleolar and trimalleolar fractures. Surprisingly, poor outcomes were found for isolated medial malleolar fractures. Development of osteoarthritis occurred mainly in trimalleolar fractures with a posterior fragment larger than 5 %.

**Conclusions:**

The results of our study show that long-term functional outcome is strongly associated to medial malleolar fractures, isolated or as part of bi- or trimalleolar fractures. More cases of osteoarthritis are found in trimalleolar fractures.

## Background

Ankle fractures are commonly seen at emergency departments, accounting for approximately 10 % of all fractures [1]. In general, stable fractures are treated with cast immobilisation, whereas unstable fractures are mainly treated by internal fixation [2, 3]. In the literature, little attention is given to the long-term functional outcome of operatively treated ankle fractures. Some studies have compared combined uni and bimalleolar fractures to trimalleolar fractures [4, 5]. Whereas other studies have focused on the long-term influence of deltoid ligamentous injury in addition to a fibular fracture, or on the role of the posterior fragment in ankle fractures [6–10]. To our knowledge, no study has compared the long-term results of operatively treated unimalleolar fractures with bimalleolar and trimalleolar fractures. In this study, we analysed the influence of the number and location of malleolar fractures on long-term function, pain, range of motion and development of osteoarthritis in 243 operatively treated participants.

## Methods

### Participants

In this retrospective cohort study we included all participants with an ankle fracture, who were operatively treated in our clinic from 1995 until 2007. Exclusion criteria were open fractures, epiphyseal fractures, pathological fractures, pilon fractures, previous ankle fracture on the same side, polytrauma patients, age under 18 years at the time of trauma and older than 75 years at the time of follow-up. No Wet Medisch Onderzoek requirement (Medical Research in Humans) was needed according to the local Medical Ethical Committee. All participants who met the inclusion criteria were invited to participate in the study. If the patient did not have a current phone number or address, the family doctor’s registry was used to obtain the current phone number or address. If patients still could not be reached, the patients were considered lost to follow-up. Patients willing to participate provided written informed consent after being informed about the study.

### Radiographic assessment and surgical procedure

**Fig. 1:**
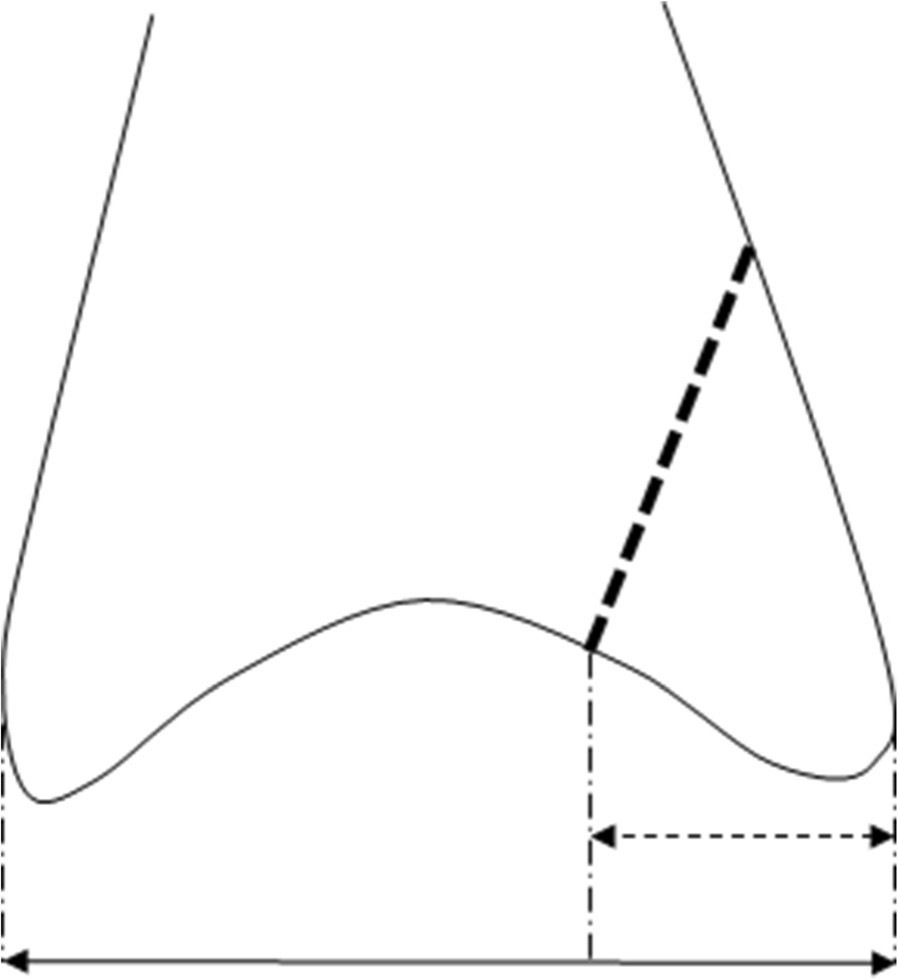
Calculation of size of the posterior fragment

All initial radiographs were grouped according to the AO and Lauge-Hansen [11] classifications by two independent observers. In addition, we grouped all fractures on the basis of the initial x-ray into the following groups based on the location and number of fractures: isolated fibular fracture (F), fibular fracture with additional posterior fracture (FP), isolated medial fracture (M), bimalleolar (combination of fibular and medial) fracture (FM) or trimalleolar fracture if a lateral, medial and posterior fracture were present (T). Dislocation of the fragments, congruency of the joint space, medial clear space in isolated fibular fractures, and the size of the posterior fragment in cases of involvement of the posterior malleolus were measured both on preoperative and postoperative X-rays. Medial clear space was measured on the mortise view as the distance between the lateral border of the medial malleolus and the medial border of the talus. A space greater than 4 mm was considered as abnormal because it indicates a lateral shift of the talus due to deltoid ligamentous injury leading to incongruency of the ankle joint [12]. The indication for surgical intervention in isolated fibular fractures without deltoid ligament injury was dislocation ≥ 2 mm of the fragments. Evaluation of syndesmotic widening was performed by measuring the distance between the medial wall of the fibula and the incisural surface of the tibia, which should be less than 6 mm both on AP and mortise views [12]. Size of posterior fragment was defined as the length of the joint-involved part of the posterior fragment divided by the total length of the joint surface in anterior-posterior direction (Fig. 1). All participants were treated surgically and fixation took place according to AO principles. Large posterior fragments were reduced closed or percutaneously and fixed by anterior-posterior placement of 1 or 2 screws under fluoroscopic control. Type of surgery depended on preference of the attending surgeon.

### Study procedure

**Fig. 2:**
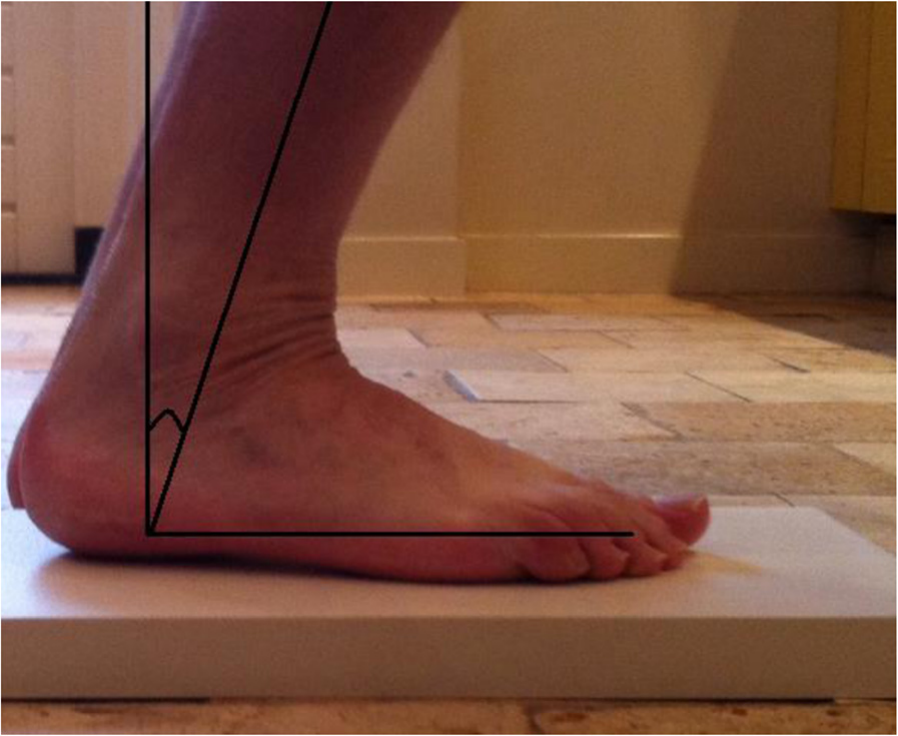
Measuring maximal dorsiflexion of the injured and healthy ankle

Baseline participant characteristics as well as data on operation delay, immobilisation duration, fixation technique and complications were obtained from the hospital records. The participants were seen at the outpatient clinic where physical examination was performed including range of motion, two questionnaires to assess functional outcome and x-rays of the injured ankle to assess osteoarthritis (mortise and lateral) were performed. Maximum dorsiflexion was measured using a goniometer after the patient had placed the affected foot on a 2.5 cm elevation (Fig. 2). This was done for both ankles and the difference was registered as dorsiflexion restriction, which is mostly limited if ankle problems exists.

Functional outcome was assessed using the American Orthopaedic Foot and Ankle Society (AOFAS) [13, 14], the American Academy of Orthopaedic Surgeons (AAOS) [15] and the VAS-pain scale. Evaluation of osteoarthritis was performed with a standardised model that grades osteoarthritis into 4 categories; Grade 1: no osteophytes, no joint space narrowing; Grade 2: small osteophytes, no joint space narrowing; Grade 3: moderate osteophytes, joint space narrowing; Grade 4: large osteophytes, severe joint space narrowing [16].

The collected data were statistically analysed with SPSS, Version 17.0. Major endpoints were function, VAS-pain, dorsiflexion restriction of the affected ankle compared to the contralateral non-fractured side and degree of osteoarthritis. Normally distributed continuous data were compared between groups with parametric tests (*t*-test or ANOVA). In case of skewed distribution in numerical data, the Mann–Whitney test or the Kruskal-Wallis test was used. For categorical data, the Fisher’s exact test or chi-squared test was used. A two-tailed *p*-value below 0.05 was considered as statistically significant.

## Results

**Fig. 3:**
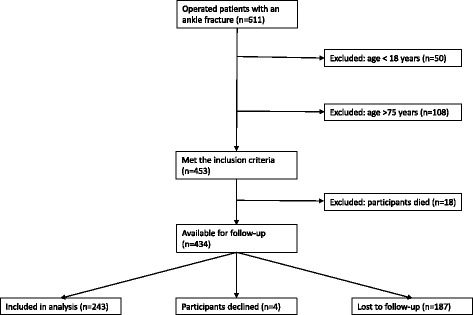
Flowchart of participants

Of the 611 patients with an operatively treated ankle fracture in the study period, 434 met the inclusion criteria and were invited to participate in the study. Of these, 243 agreed to participate and were evaluated between January and May 2012 (Fig. 3). The average age of the participants was 52 years at the time of evaluation after a mean follow-up of 9.6 years (range 5–17 years). One hundred and fourteen participants (47 %) were men; mean Body Mass Index at time of evaluation was 28.0 kg/m^2^. According to the radiographic classification, the study population consisted of 112 participants with an isolated fibular fractures (group F), 20 participants with a combination of fibular and posterior malleolar fracture (group FP), 9 participants with an isolated medial fracture (group M), 43 participants with bimalleolar fractures (group FM), and 59 participants with trimalleolar fractures (group T). Baseline characteristics of the groups are presented in Table 1. The mean size of the posterior fragment was 16 % (range, 3–53 %). The mean time between trauma and internal fixation (operation delay) was 5 days. Postoperatively, all x-rays showed proper reduction (less than 2 mm dislocation) and a good joint congruency. Fixation of the posterior fragment took place in 11 cases: 8 fragments were larger than 25 % of the involved articular surface, and 3 were between 5–25 %.

**Table 1:** Participant characteristics

Group	*N*	Age (y)	Follow-up (y)	BMI	Posterior fragment (%)	Male (%)	Diabetes (%)	Smoking (%)
Total	243		9.7	28.0		47 %		
F	112	52	9.8	27.8		48 %	15 %	33 %
FP	20	48	8.6	29.1	13.0	40 %	0 %	47 %
M	9	43	10.9	26.2		67 %	13 %	50 %
FM	43	53	10.1	28.6		51 %	18 %	24 %
T	59	55	9.2	27.2	16.7	41 %	13 %	25 %

Nineteen complications occurred in 19 (7.8 %) participants, mainly in those with trimalleolar fractures. In the total population, mechanical or technical problems (loose screws) occurred in 5 cases, reoperation was necessary in 3 participants (all because of secondary widening of the syndesmosis), and superficial or deep infection occurred in 14 participants (all participants were sufficiently treated by oral antibiotics). Talocrural arthrodesis (2 participants) or ankle-prosthesis (1 participant) was necessary due to severe post-traumatic osteoarthritis. Due to pain or discomfort removal of the implants took place in 70 (29 %) participants after 13 months on average.

The median scores of the outcome measures in this population are presented in Table 2. Our data show that there was no significant difference between fibular fractures with or without additional deltoid ligamentous injury (AOFAS 98 vs 95, *p* = 0.255; AAOS 96 vs 97, *p* = 0.497; VAS 1.0 vs 1.1, *p* = 0.064, dorsiflexion-restriction 3.3° vs 1.8°, *p* = 0.221). An additional fracture of the posterior malleolus in fibular fractures leads to comparable outcome (Table 2; AOFAS 97 vs 97, *p* = 0.801; AAOS 97 vs 98, *p* = 0.772; VAS 1.1 vs 1.0, *p* = 0.990; dorsiflexion restriction 2.8° vs 3.3°, *p* = 0.565). Significant differences were found between isolated fibular and bimalleolar fractures, except for VAS-pain, (Table 2; AOFAS 97 vs 91, *p* = 0.035; AAOS 97 vs 90, *p* = 0.026; VAS 1.1 vs 1.8, *p* = 0.263; dorsiflexion restriction 2.8° vs 6.7°, *p* = 0.003) and between isolated fibular and trimalleolar fractures (AOFAS 97 vs 88, *p* < 0.001; AAOS 97 vs 90, *p* = 0.003; VAS 1.1 vs 2.3 *p* < 0.001; dorsiflexion restriction 2.9° vs 6.9°, *p* < 0.001).

**Table 2:** Overview of study results

Group	*N*	AAOS	AOFAS	VAS-pain	Dorsiflexion-restriction	OA grade 1	OA grade 2	OA grade 3	OA grade 4
Total	243	95	95	1.5	4.6°	205	20	14	4
F	112	97	97	1.1	2.8°	104	5	3	0
FP	20	97	98	1.0	3.3°	16	3	1	0
M	9	89	83	2.5	5.0°	9	0	0	0
FM	43	90	91	1.8	6.7°	39	2	2	0
T	59	90	88	2.3	69°	45	10	8	4

Legend: F = isolated fibular fracture, FP = fibular fracture with additional posterior fracture, M = isolated medial fracture, FM = bimalleolar (combination of fibular and medial) fracture, T = trimalleolar fracture

Fibular fractures with additional posterior fragment had a significantly better functional outcome than trimalleolar fractures (Table 2; AOFAS 97 vs 88, *p* = 0.040; AAOS 98 vs 90, *p* = 0.011; VAS 1.0 vs 2.3, *p* = 0.006; dorsiflexion restriction 3.3° vs 6.9°, *p* = 0.034). The size of the posterior fragment did not differ significantly between these two groups (respectively 13 and 17 %). No significant differences were found between bimalleolar and trimalleolar fractures on all outcomes (Table 2; AOFAS 91 vs 88, *p* = 0.180; AAOS 90 vs 90, *p* = 0.220; VAS-pain 1.8 vs 2.3, *p* = 0.191; dorsiflexion restriction 6.7° vs 6.9°, *p* = 0.822). Isolated medial fractures had surprisingly bad results. However, due to the small group size there was no significant difference compared to the other groups.

All fractures with a posterior fragment were further subdivided by size of the posterior fragment; group 1 consisted of fragments <5 %, group 2 of fragments between 5 and 25 % and group 3 of fragments > 25 % of involved articular surface (Table 3). We found that fragments larger than 5 % resulted in a worse functional outcome than fragments smaller than 5 %, although this difference was not statistically significant.

**Table 3:** Results in participants with a posterior malleolar fracture

Size of posterior fragment	*N*	AOFAS	VAS-pain	Dorsiflexion-restriction	OA grade 1	OA grade 2	OA grade 3	OA grade 4
<5 %	8	95	1.4	2.9°	8	0	0	0
5–25 %	56	88	2.1	6.2°	38	9	7	2
>25 %	14	90	1.6	7.8°	10	1	1	2

Some degree of osteoarthritis was found in 8 (7 %) unimalleolar fibular fractures, 4 (20 %) fibular fractures with additional posterior malleolar fracture, 4 (9 %) bimalleolar fractures and 22 (37 %) trimalleolar fractures (Table 2). Grade 2 osteoarthritis was present in 20 cases, grade 3 in 14 cases, and grade 4 in only 4 cases (all of whom had trimalleolar fractures). The 3 participants with an ankle-prosthesis or arthrodesis were included in this analysis and all were part of the trimalleolar group. In fractures with involvement of the posterior tibial margin, osteoarthritis was only found in medium-sized (33 %) and large-sized posterior fragments (29 %) (Table 3).

To compare our results with other publications, we also classified the participants according to the AO and Lauge-Hansen classifications. No significant differences between the 3 main groups of the AO-classification were found (Table 4). Likewise, no significant differences were found between the 4 main-groups of the Lauge-Hansen classification (Table 5).

**Table 4:** Results in total population classified according the AO-classification

	*N*	AAOS	AOFAS	VAS-pain	Dorsiflexion-restriction
AO 44-A	6	99	95	0.7	5.0
AO 44-B	168	96	95	1.4	4.2
AO 44-C	61	94	94	1.8	5.6

**Table 5:** Results in total population classified according the Lauge-Hansen classification

	*N*	AAOS	AOFAS	VAS-pain	Dorsiflexion-restriction
Supination-adduction	6	94	95	1.5	5.0
Supination-external rotation	149	95	95	1.5	3.6
Pronation-abduction	26	96	96	0.9	7.0
Pronation-external rotation	62	94	94	1.8	5.9

## Discussion

The majority of the participants in this, as far as we know, the largest long-term cohort study of operated ankle fractures showed good results after a mean follow-up of nearly 10 years. In contrast to earlier studies, this study compares large subgroups of operatively treated ankle fractures on long-term outcome where other study groups were small groups or had a short follow-up period.

Previous long-term studies mainly focus on trimalleolar fractures, and especially on the influence of the size of the involved articular surface, and whether or not the posterior fragment should be fixed [7–9]. In other articles, a combined group of uni and bimalleolar fractures are compared to trimalleolar fractures [4, 5]. In 1989, Jaskulka et al. were the first authors to compare the long-term outcome of uni and bimalleolar fractures with trimalleolar fractures [4]. They described a significantly worse outcome in trimalleolar fractures. They also found a worse outcome on function, development of osteoarthritis and pain in trimalleolar fractures with a posterior fragment larger than 5 % compared to posterior fragments smaller than 5 % of the involved articular surface. Limitations of this study are the relatively short follow-up period (5.7 years) and the fact that unimalleolar and bimalleolar fractures were not analysed separately. In a prospective cohort-study with a follow-up period of one year, Tejwani et al. also described a worse outcome of trimalleolar fractures compared to uni and bimalleolar fractures [10]. A small part of that cohort, which was followed for 2 years, showed decreasing differences between the two groups and therefore, the authors concluded that there is no difference between long-term outcome of uni, bi or trimalleolar fractures.

In this study, analysis of isolated fibular fractures was conducted in two subgroups: with or without suspicion of an additional ruptured deltoid ligament (if the medial clear space was >4 mm). There were no significant differences between these subgroups. Therefore, we agree with Donken et al. that additional deltoid ligament injury does not lead to a worse functional outcome in the long-term in operatively treated fibular fractures [6].

Surprisingly, the outcomes of fibular fractures with an additional posterior fragment were similar to isolated fibular fractures. If bimalleolar fractures are compared with trimalleolar fractures there is no significant difference in functional outcome, except for pain. However, a fracture of the medial malleolus is, according to our data, strongly associated with worse functional outcome.

Despite the large cohort, this study did not show a clear and significant relation between size of the posterior fragment and long-term functional outcome in trimalleolar fractures. Our results suggest that a posterior fragment larger than 5 % leads to a worse outcome compared to posterior fragments smaller than 5 % of the involved articular surface. This is in accordance with Jaskulka et al. [4]. Langenhuijsen et al. described a worse outcome if the posterior fragment is larger than 10 % of the involved articular surface [8]. De Vries et al., Mingo-Robinet et al. and Xu et al. found no clear relationship between posterior fragment size or fixation of the posterior fragment and long-term functional outcome [7, 9, 17].

To clarify these inconsistent findings, we are currently expanding our study with a more recently operated cohort of participants with trimalleolar fractures. Due to the small sample size of the present study, we cannot draw any conclusions with respect to fixation of posterior fragments and the influence on long-term functional outcome as yet.

Unimalleolar and bimalleolar fractures lead to osteoarthritis in only a small percentage of cases. Osteoarthritis in this type of fractures is mainly caused by other factors such as screw displacement or infection. In our series, osteoarthritis occurred mainly in trimalleolar fractures and is therefore thought to be a result of the combination of a posterior malleolar fracture and a medial malleolar fracture. In trimalleolar fractures, the initial cartilage damage in the weight-bearing part of the joint, in combination with the increased peak contact stress and the weight-bearing shift to anterior and medial, with a non-anatomical reduction of the posterior fragment could lead to an increase in development of osteoarthritis [18–22].

A limitation of this study is the 56 % response rate. This study was completed in an inner-city hospital with a poor patient compliance and a highly variable patient population (seasonal workers and several different nationalities). Despite maximum effort to trace these patients many potential participants were lost to follow-up, so there is a risk of selection bias. However, the baseline data and fracture characteristics of the study group were similar to the total group of patients who met the inclusion criteria for the study. This indicates that our study group was representative.

## Conclusions

Our data support the assumption that in operated isolated fibular fractures, an additional rupture of the deltoid ligament does not lead to worse outcome. Moreover, the results of our study show that long-term functional outcome is strongly associated to the medial malleolus. Bimalleolar fractures lead to significantly worse functional results than isolated fibular fractures and are comparable to trimalleolar fractures. In case of a posterior fracture, the involvement of the medial malleolus will lead to worse functional outcome, more pain and more development of osteoarthritis.

## References

[1] Court-Brown CM, McBirnie J, Wilson G (1998). Adult ankle fractures--an increasing problem?. Acta Orthop Scand.

[2] Van den Bekerom MP, Lamme B, Hogervorst M, Bolhuis HW (2007). Which ankle fractures require syndesmotic stabilization?. J Foot Ankle Surg.

[3] Van den Bekerom MP, Haverkamp D, Kerkhoffs GM, van Dijk CN (2010). Syndesmotic stabilization in pronation external rotation ankle fractures. Clin Orthop Relat Res.

[4] Jaskulka RA, Ittner G, Schedl R (1989). Fractures of the posterior tibial margin: their role in the prognosis of malleolar fractures. J Trauma.

[5] McDaniel WJ, Wilson FC (1977). Trimalleolar fractures of the ankle. An end result study. Clin Orthop Relat Res.

[6] Donken CC, Verhofstad MH, Edwards MJ, van Laarhoven CJ (2012). Twenty-one-year follow-up of supination-external rotation type II-IV (OTA type B) ankle fractures: a retrospective cohort study. J Orthop Trauma.

[7] De Vries JS, Wijgman AJ, Sierevelt IN, Schaap GR (2005). Long-term results of ankle fractures with a posterior malleolar fragment. J Foot Ankle Surg.

[8] Langenhuijsen JF, Heetveld MJ, Ultee JM, Steller EP, Butzelaar RM (2002). Results of ankle fractures with involvement of the posterior tibial margin. J Trauma.

[9] Mingo-Robinet J, Lopez-Duran L, Galeote JE, Martinez-Cervell C (2011). Ankle fractures with posterior malleolar fragment: management and results. J Foot Ankle Surg.

[10] Tejwani NC, Pahk B, Egol KA (2010). Effect of posterior malleolus fracture on outcome after unstable ankle fracture. J Trauma.

[11] Yde J (1980). The Lauge Hansen classification of malleolar fractures. Acta Orthop Scand.

[12] Marsh JL, Saltzman CL, Rockwood and Green’s (2013). Ankle fractures. Fractures in adults.

[13] SooHoo NF, Shuler M, Fleming LL (2003). Evaluation of the validity of the AOFAS Clinical Rating Systems by correlation to the SF-36. Foot Ankle Int.

[14] Ibrahim T, Beiri A, Azzabi M, Best AJ, Taylor GJ, Menon DK (2007). Reliability and validity of the subjective component of the American Orthopaedic Foot and Ankle Society clinical rating scales. J Foot Ankle Surg.

[15] Johanson NA, Liang MH, Daltroy L, Rudicel S, Richmond J (2004). American Academy of Orthopaedic Surgeons lower limb outcomes assessment instruments. Reliability, validity, and sensitivity to change. J Bone Joint Surg Am.

[16] Domsic ST (1998). Ankle Osteoarthritis Scale. Foot Ankle Int.

[17] Xu HL, Li X, Zhang DY, Fu ZG, Wang TB, Zhang PX (2012). A retrospective study of posterior malleolus fractures. Int Orthop.

[18] Vrahas M, Fu F, Veenis B (1994). Intraarticular contact stresses with simulated ankle malunions. J Orthop Trauma.

[19] Hartford JM, Gorczyca JT, McNamara JL, Mayor MB. Tibiotalar contact area. Contribution of posterior malleolus and deltoid ligament. Clin Orthop Relat Res. 1995;182–7. 7586825

[20] Harper MC. Talar shift. The stabilizing role of the medial, lateral, and posterior ankle structures. Clin Orthop Relat Res. 1990;177–83. 2379358

[21] Fitzpatrick DC, Otto JK, McKinley TO, Marsh JL, Brown TD (2004). Kinematic and contact stress analysis of posterior malleolus fractures of the ankle. J Orthop Trauma.

[22] Macko VW, Matthews LS, Zwirkoski P, Goldstein SA (1991). The joint-contact area of the ankle. The contribution of the posterior malleolus. J Bone Joint Surg Am.

